# Effects of Metformin on Renal Function, Cardiac Function, and Inflammatory Response in Diabetic Nephropathy and Its Protective Mechanism

**DOI:** 10.1155/2022/8326767

**Published:** 2022-06-03

**Authors:** Zhiping Zhang, Hongyu Dong, Jiaqi Chen, Min Yin, Feng Liu

**Affiliations:** ^1^Department of Nephrology, China-Japan Union Hospital of Jilin University, Changchun, China; ^2^Department of Rheumatology and Immunology, Shijingshan Teaching Hospital of Capital Medical University, Beijing Shijingshan Hospital, Beijing 100043, China

## Abstract

**Objective:**

To investigate the effect of metformin on renal function, cardiac function, and inflammatory response in diabetic nephropathy and its protective mechanism.

**Methods:**

A total of 88 patients with diabetic nephropathy who were admitted to our hospital from April 2019 to October 2020 were recruited and grouped according to different treatment methods, namely, the experimental group (*n* = 44) and the control group (*n* = 44). The patients in the experimental group were treated with metformin, and the patients in the control group were treated with liraglutide injection (nonmetformin). Left ventricular end-diastolic diameter (LVEDD), left ventricular ejection fraction (LVEF), left ventricular end-systolic diameter (LVESD), and inflammatory response (hs-CRP, TNF-*α*, IL-6) were compared.

**Results:**

Compared with corresponding values before treatment, BUN, Scr, hs-CRP, TNF-*α*, IL-6, LVEDD, and LVESD were decreased after treatment, whereas LVEF was increased (all *P* < 0.05), with significant change in the experimental group (all *P* < 0.001).

**Conclusion:**

Metformin can effectively improve the level of renal function and cardiac function in patients with diabetic nephropathy and help patients control and reduce the body's inflammatory response, and its therapeutic efficacy is superior to that of liraglutide injection.

## 1. Introduction

Type 2 diabetes is one of the endocrine diseases characterized by elevated blood sugar, which is ascribed to impaired biological effects of the human body or defective insulin secretion. Recent years witness a rising incidence of type 2 diabetes, with the improvement of people's living standards. Delayed and ineffective treatment gives rise to complications of the heart, kidneys, and other organs, among which diabetic nephropathy is of higher occurrence and is associated with renal failure. Consequently, early intervention is required to curb the development of the disease [1, 2]. Diabetic hyperglycemia may either directly induce nephropathy or indirectly induce nephropathy by altering hemodynamics and also induce protein kinase C (PKC) activity, increase glycation end products, and promote the generation of triacylglycerol. In addition, hyperglycemia can also lead to changes in hemodynamics, causing glomerular filtration, shear stress, and microalbuminuria. These changes stimulate resident kidney cells to produce more TGF-*β*1, which in turn downregulates glucose transporter 1 and increases intracellular glucose transport and D-glucose uptake. TGF-*β*1 causes excessive accumulation of interstitial proteins (collagens I, II, III, and IV; fibronectin; and laminin) in the glomerulus, resulting in mesangial expansion and thickness of glomerular basement membrane and thereby changing the structure of the nephron. At present, the incidence of nephropathy in type 2 diabetic patients in China is as high as 30-50%. It is worth noting that there remains no cure to delay the progression of diabetic nephropathy. Additionally, a US study revealed a higher prevalence of diabetic nephropathy despite the effective control of cardiovascular complications, imposing substantial health care and economic burden. Liraglutide is a long-acting glucagon-like peptide 1 (GLP-1) analog to lower blood sugar and inhibit glucagon secretion by promoting insulin release. Metformin is one of the commonly used drugs for the treatment of diabetes, and its main mechanism of action is to improve insulin sensitivity by inhibiting intestinal absorption of glucose, increase glucose utilization, and then lower blood sugar levels, with good safety profile [3, 4]. However, there are few related studies on its impact on renal function, cardiac function, and inflammatory response and its protective mechanism [5–7]. Accordingly, the principal aim of the present study was to explore the effects of metformin on renal function, cardiac function, and inflammatory response in diabetic nephropathy and its protective mechanism.

## 2. Materials and Methods

### 2.1. Baseline Data

A total of 88 patients with diabetic nephropathy who were treated in our hospital from April 2019 to October 2020 were assigned into groups according to different treatment methods, namely, the experimental group (*n* = 44) and the control group (*n* = 44). The study protocol was approved by the Ethics Committee of China-Japan Union Hospital of Jilin University (approval no. 60301-198), and all the subjects signed the informed consent and voluntarily participated in the clinical trial. In the experimental group, there were 24 males and 20 females; aged between 60 and 80 years; the disease duration was between 5 and 14 years. In the control group, there were 23 males and 21 females; the age ranged from 60 to 79 years, and the disease duration was between 5 and 14 years. The baseline data were similar in the two groups (Table 1).

### 2.2. Inclusion and Exclusion Criteria

The participants were eligible if they met the following criteria: (1) met the diagnostic criteria for diabetic nephropathy; (2) had complete medical data; (3) had normal cognition and good coordination; (4) aged 18-70 years; (5) used stable dose of metformin for more than 3 months; (6) HbA1c 7.0%-10.0%, no significant change in body weight at least 12 weeks before screening; (7) showing left ventricular ejection fraction (LVEF) > 50% by echocardiography; and (8) agreed to continue to maintain the previous diet and exercise habits throughout the study process, not using antihypertensive drugs such as statin lipid-lowering drugs and angiotensin-converting enzyme inhibitor (ACEI)/angiotensin II receptor antagonists during treatment (ARB). Patients were assessed as ineligible if they (1) had mental disorder; (2) had heart, liver, and renal failure; (3) received other clinical trials recently; (4) withdrew from the study; (5) had chronic complications of diabetes with notable clinical significance, such as proliferative retinopathy; (6) have had or are currently suffering from ischemic cardiovascular and cerebrovascular disease or peripheral vascular disease; (7) had systolic blood pressure > 160 mm within the last 12 weeks before screening Hg (1 mm Hg = 0.133 kPa) and/or diastolic blood pressure>100 mm Hg; (8) had a history of pancreatic or thyroid disease; and (9) had type 2 diabetes mellitus complicated with pregnancy.

### 2.3. Methods

Both groups of patients received medication for 4 months, with strict diet and exercise management during the period. The patients in the experimental group were treated with metformin (approval no. H20023370, specification: 0.5 g/tablet), 1 tablet/time, 3 times/d. The patients in the control group were subcutaneously injected with liraglutide injection (produced by Novo Nordisk, Denmark, approval no. J20160037, specification: 3 ml: 18 mg), 0.6 g/time, 1 time/d.

### 2.4. Outcomes

#### 2.4.1. Renal Function Indicators

Blood urea nitrogen (BUN) and serum creatinine (Scr) were detected in the two groups of patients. The venous blood was collected and centrifuged at a speed of 1000 r/min and a radius of 10 cm for 5 min, and then, the supernatant was secured. The urea nitrogen (BUN) and serum creatinine (Scr) values were detected by an automatic biochemical method. The detection kit and the supporting kit were purchased from Nanjing Biyuntian Biological Testing Company, and the microcentrifuge HITETIC was purchased from Shanghai Precision Instrument Co., Ltd.

#### 2.4.2. Cardiac Function Indicators

The color Doppler ultrasound diagnostic instrument purchased from Beijing Beiden Medical Co., Ltd. was used to detect the cardiac function indicators of patients, including left ventricular end-diastolic diameter (LVEDD), left ventricular end-systolic diameter (LVESD), left ventricular end-diastolic diameter (LVESD), left ventricular end-diastolic diameter (LVESD), and ventricular ejection fraction (LVEF).

#### 2.4.3. Inflammatory Response Indicators

3 mL of venous blood was collected from patients before and after treatment, and high-sensitivity C-reactive protein (hs-CRP), tumor necrosis factor-*α* (TNF-*α*), and interleukin-6 (IL-6) monoclonal antibodies were added. Monoclonal antibody was mixed and washed with PBS for 5 min. According to the ratio of 1 : 500, 5 mL of human-sheep-labeled primary antibody was added and left overnight at 4°C; then, it was washed 3 times with PBS buffer, 5 min each time, and 2 mL of mouse-derived secondary antibody (1 : 1000) was added; finally, it was left at room temperature for 2 hours and washed with PBS buffer for 5 minutes. The chromogenic substrate horseradish peroxidase was added to make the colorless chromogenic reagent blue, and the stop buffer was added to make it yellow. OD values were measured at 450 nm.

### 2.5. Statistical Analysis

All data analyses were performed with SPSS22.0. The measurement data are expressed as *x* ± *s*, and two independent sample *t*-tests were used for comparison between groups, and a paired t-test was used for comparison within groups; the count data are expressed as the number of cases (rate) and analyzed by a chi-square test. Statistical significance was set at *P* < 0.05.

## 3. Results

### 3.1. Renal Function

There was no significant difference in BUN and Scr between the two groups before treatment (*P* > 0.05). After treatment, BUN and Scr were lower than the corresponding values before treatment (*P* < 0.05); the decrease was greater in the experimental group as compared with the control group [BUN (8.63 ± 2.07) and Scr (72.42 ± 16.78) vs. BUN (12.26 ± 2.93) and Scr (89.51 ± 23.26)] (*P* < 0.001); see Table 2.

### 3.2. Cardiac Function

There was no significant difference in LVEDD, LVEF, and LVESD between the two groups before treatment (*P* > 0.05). After treatment, in the experimental group, LVEDD was53.27 ± 1.02, LVEF was53.51 ± 1.24, and LVESD was51.15 ± 0.65, and in the control group, LVEDD was56.02 ± 1.39, LVEF was49.06 ± 1.37, and LVESD was54.13 ± 1.13. Overall, LVEDD and LVESD were decreased, and LVEF was increased in both groups (*P* < 0.05) after treatment, with greater change in the experimental group (*P* < 0.001); see Table 3.

### 3.3. Comparison of Inflammatory Responses

There was no significant difference in hs-CRP, TNF-*α*, and IL-6 between the two groups before treatment (*P* > 0.05); after treatment, in the experimental group, hs-CRP was9.08 ± 1.12, TNF-*α* was14.51 ± 1.24, and IL-6 was11.15 ± 1.45; in the control group, hs-CRP was11.14 ± 1.19, TNF-*α* was19.06 ± 1.37, and IL-6 was16.84 ± 1.22. Overall, hs-CRP, TNF-*α*, and IL-6 were lower than the corresponding values before treatment (*P* < 0.05), with lower results in the experimental group (*P* < 0.001), as shown in Table 4.

## 4. Discussion

The main clinical manifestations of diabetic patients include polyphagia, polydipsia, polyuria, and weight loss. Delayed treatment might lead to metabolic disorders such as abnormal amount of carbohydrates, electrolytes, proteins, and fats, impairing organs such as the kidneys and resulting in diabetic nephropathy that can cause renal failure. As a result, early intervention is an urgent to prevent the development of the disease [8, 9]. In severe cases, diabetic nephropathy can even cause death and serves as the main contributor to death in patients with diabetes [10–12]. Metformin has been confirmed to be an effective alternative in early diabetic nephropathy [13, 14]. To our knowledge, the nephroprotective effect of metformin is closely related to its inhibition of Adenosine Monophosphate-Activated Protein Kinase (AMPK)/mammalian target of rapamycin (mTOR) signaling pathway. DeFronzo et al. [15] believed that metformin, as a first-line oral hypoglycemic drug, not only acts as a hypoglycemic agent but also acts as an AMPK activator, intervening in the pathological development process of the abovementioned diabetic nephropathy by activating the AMPK signaling pathway, thereby exerting a renal protective effect and delaying the diabetic nephropathy development; Klotho is an antiaging gene, and its expressed klotho protein is produced in the distal convoluted tubules of the kidney. With the progression of kidney disease, the expression of klotho protein in patients with diabetic nephropathy decreases, and the decrease of klotho can activate the mTOR signaling pathway and aggravate kidney damage. In this study, we compared the renal function indexes of the patients in the experimental group before and after treatment. The results showed that after treatment, BUN and Scr were lower than the corresponding values before treatment; the decrease was greater in the experimental group as compared with the control group. These findings suggest that metformin is effective in the improvement of renal function. The possible explanation is that metformin regulates the mTOR pathway by upregulating klotho and protects renal tubular cells, thereby delaying renal progression in patients with diabetic nephropathy. Diabetic nephropathy is a chronic low-grade inflammatory disease, and diabetic nephropathy and inflammatory response were closely correlated. CRP, one of the acute response phase proteins, can reflect the level of inflammation in the body and the progress of cerebrovascular disease; TNF-*α* and IL-6 are proinflammatory factors, both of which can promote the chemotaxis and adhesion of inflammatory factors, and adversely affect patients. Notably, we found that the inflammatory response (hs-CRP, TNF-*α*, and IL-6) of the patients in the experimental group was declined after treatment, indicating that metformin can effectively reduce the inflammatory response of the patients. It might be attributed to the fact that metformin can enhance insulin resistance, regulate blood lipids and blood pressure, and effectively reduce the chemotaxis and adhesion of inflammatory factors [15–18]. Similarly, several studies concluded that metformin dose-dependently upregulated catalase (CAT), NADPH quinone oxidoreductase (NQO1) and glutathione S-transferase (GST*α*) mRNA expression in renal tissue, decreased Heme oxygenase 1 (HO-1), TNF-*α*, IL-6 mRNA expression, and transforming growth factor in blood *β*1 (TGF-*β*1) levels, indicating that metformin exerts renal protection by reducing the levels of renal oxidative stress, inflammation, and fibrosis [17, 18]. Liu et al. [13] also found that metformin reduced GRP78, eIF2*α*, and C/EBP homologous protein (CHOP) by activating AMPK and attenuated high glucose-induced oxidative stress in human proximal tubular epithelial cells. Mariano and Biancone [14] found that metformin activates AMPK, reduces the production of RAGE and ROS, and further reduces the expression of its downstream signal TGF-*β*1, thereby inhibiting tubular fibrosis. Since a number of studies have been conducted on the drug mechanism of metformin and are relatively complete, this study innovatively increased the discussion of cardiac function indicators on this basis. Relevant studies have shown that dimethicone has a good protective effect on the cardiovascular system, and the 2016 *European Heart Failure Guidelines* recommended dimethicone as the first-line drug for patients with diabetes and heart failure. Promisingly, we observed in the present study that the cardiac function indexes of the patients in the experimental group were significantly improved after treatment, suggesting that metformin could effectively improve the cardiac function of the patients such as LVEDD, left LVEF, and LVESD. Presumably, metformin reduces endothelial cell oxidative stress by improving insulin resistance, blood sugar, blood lipids, and other cardiovascular-related risk factors, thereby protecting vascular endothelial cell function and further reduce the occurrence of cardiovascular disease. Additionally, it can promote myocardial glucose uptake and further improve the insulin sensitivity, thereby reducing myocardial cell damage and boosting cardiac function [19–21].

In conclusion, metformin is a reliable drug to improve the renal function and cardiac function of patients with diabetic nephropathy and helps patients control and minimize inflammatory response, which is superior to liraglutide injection.

## Figures and Tables

**Table 1:** Patients' characteristic profile (*x* ± *s*).

Index	Experimental group (*n* = 44)	Control group (*n* = 44)	*t*	*P*
Average age (years)	66.42 ± 3.72	66.10 ± 3.69	1.441	0.531
Average disease duration (years)	7.85 ± 2.11	7.62 ± 2.01	2.475	0.634
Urine albumin/creatinine ratio (ACR, mg/g)	280.47 ± 33.54	281.21 ± 32.69	5.245	0.548
Fasting blood glucose (mmol/L)	7.75 ± 2.40	7.76 ± 2.38	3.564	0.685
HbA1c (%)	8.54 ± 2.06	8.48 ± 2.11	2.475	0.365
Triglycerides (TG, mmol/L)	2.49 ± 0.72	2.47 ± 0.68	1.454	0.254
Total cholesterol (TC, mmol/L)	4.75 ± 1.03	4.72 ± 1.10	3.457	0.541
Low-density lipoprotein cholesterol (LDL-C, mmol/L)	3.42 ± 0.91	3.38 ± 0.89	5.456	0.477
High-density lipoprotein cholesterol (HDL-C, mmol/L)	1.12 ± 0.21	1.14 ± 0.19	1.045	0.698
Serum uric acid (mmol/L)	360.52 ± 91.89	360.61 ± 92.87	3.745	0.371
AST (U/L)	28.58 ± 11.64	28.21 ± 10.94	2.475	0.654
ALT (U/L)	26.45 ± 11.14	26.78 ± 12.36	2.315	0.638
Glomerular filtration rate (GFR [ml^−1^ min^−11^ (1.73 m^2^)^−11^])	78.13 ± 26.11	77.87 ± 25.96	1.457	0.638

**Table 2:** Comparison of renal function indexes of patients (*x* ± *s*).

Groups	*n*	BUN (mmol/L)		Scr (*μ*mol/L)	
		Before treatment	After treatment	Before treatment	After treatment
Study group	44	16.33 ± 3.18	8.63 ± 2.07^∗^	92.24 ± 18.08	72.42 ± 16.78^∗^
Control group	44	16.28 ± 3.15	12.26 ± 2.93^∗^	92.31 ± 17.99	89.51 ± 23.26^∗^
*t*	—	0.074	-6.712	-0.018	-3.953
*P*	—	0.941	<0.001	0.986	<0.001

Note: compared with before treatment within the same group, ^∗^*P* < 0.05.

**Table 3:** Comparison of cardiac function indexes of patients (*x* ± *s*).

Groups	*n*	LVEDD (mm)		LVEF (%)		LVESD (mm)	
		Before treatment	After treatment	Before treatment	After treatment	Before treatment	After treatment
Study group	44	59.46 ± 3.08	53.27 ± 1.02^∗^	47.44 ± 4.08	53.51 ± 1.24^∗^	58.50 ± 2.09	51.15 ± 0.65^∗^
Control group	44	59.39 ± 3.05	56.02 ± 1.39^∗^	47.51 ± 3.99	49.06 ± 1.37^∗^	58.49 ± 2.05	54.13 ± 1.13^∗^
*t*	—	0.107	-10.58	-0.081	15.974	0.023	-15.163
*P*	—	0.915	<0.001	0.936	<0.001	0.982	<0.001

Note: compared with before treatment within the same group, ^∗^*P* < 0.05.

**Table 4:** Comparison of inflammatory responses in patients (*x* ± *s*).

Groups	*n*	hs-CRP (mg/L)		TNF-*α* (ng/L)		IL-6 (pg/mL)	
		Before treatment	After treatment	Before treatment	After treatment	Before treatment	After treatment
Study group	44	15.07 ± 0.72	9.08 ± 1.12^∗^	27.44 ± 1.41	14.51 ± 1.24^∗^	25.87 ± 2.14	11.15 ± 1.45^∗^
Control group	44	15.05 ± 0.74	11.14 ± 1.19^∗^	27.51 ± 1.39	19.06 ± 1.37^∗^	25.93 ± 2.17	16.84 ± 1.22^∗^
*t*	—	0.128	-8.362	-0.235	-16.333	-0.131	-19.918
*P*	—	0.898	<0.001	0.815	<0.001	0.896	<0.001

Note: compared with before treatment within the same group, ^∗^*P* < 0.05.

## Data Availability

The datasets used during the present study are available from the corresponding author upon reasonable request.
